# Pyrin

**Published:** 1860-05

**Authors:** 


*Pyrin.*—We have received of Dr. J. B. Kepler, of Cerro Gordo, Indiana, a fine sample of this valuable alkaloid, the active principle of the Pyrus Dalus, or apple tree. The Dr. states that he has used this article extensively as an antiperiodic in agues and other forms of fever, and finds it as reliable as quinine. Also that it seems to possess some alterative effects upon the liver. I have used this article, and can fully corroborate the above statements relative to its medicinal virtues. Besides its antiperiodic and hepatic properties, it acts most specifically as a vermifuge. In two cases of febrile disease its administration was followed by the expulsion of ascarides or seat worms. Those of our physicians who may wish a supply of this article can obtain it by addressing Dr. Kelper as above.—*Dclectic Medical Journal.*

